# Early-life lung function deficits partially explain the link between maternal asthma and bronchiolitis or asthma in offspring

**DOI:** 10.1136/bmjresp-2025-003679

**Published:** 2026-05-18

**Authors:** Carla Rebeca Da Silva Sena, Adam Collison, Vanessa E Murphy, Gabriela Martins Costa Gomes, Olga Gorlanova, Noëmi Künstle, Céline Rüttimann, Sven Schulzke, Benjamin Stoecklin, Jakob Usemann, Ruth Steinberg, Sophie Yammine, Paul D Robinson, Peter D Sly, Philipp Latzin, Peter G Gibson, Urs Frey, Joerg Mattes

**Affiliations:** 1 University Children’s Hospital Basel UKBB, University of Basel, Basel, Switzerland; 2 Hunter Medical Research Institute and Asthma & Breathing research program, The University of Newcastle, Newcastle, New South Wales, Australia; 3 Department of Paediatrics, Inselspital University Hospital Bern, Division of Paediatric Respiratory Medicine and Allergology, Bern, Switzerland; 4 School of Medicine and Public Health, College of Health, Medicine and Wellbeing, The University of Newcastle, Newcastle, New South Wales, Australia; 5 Department of Respiratory Medicine, The Children’s Hospital at Westmead, Sydney, New South Wales, Australia; 6 South Brisbane, Children’s Health and Environment Program, Child Health Research Centre, The University of Queensland, Brisbane, Queensland, Australia; 7 Discipline of Paediatrics and Child Health, University of Sydney, Sydney, New South Wales, Australia; 8 Respiratory & Sleep Medicine Department Newcastle, John Hunter Hospital, New Lambton Heights, New South Wales, Australia; 9 Paediatric Respiratory & Sleep Medicine Department, John Hunter Children’s Hospital, Newcastle, New South Wales, Australia

**Keywords:** Respiratory Function Test, Paediatric asthma, Asthma, Asthma Epidemiology

## Abstract

**Background:**

Maternal asthma during pregnancy is associated with an increased risk of bronchiolitis and asthma in offspring, but the mechanisms remain poorly understood. We aimed to assess whether impaired postnatal infant lung function independently influences the risk of bronchiolitis and asthma in childhood or whether it mediates the association between maternal asthma during pregnancy and respiratory outcomes.

**Methods:**

We analysed harmonised infant lung function data from two birth cohorts: the Australian Breathing for Life Trial and the Swiss Basel-Bern Infant Lung Development. Bronchiolitis hospitalisation (verified through medical records) and asthma at age six (parent-reported) were primary outcomes. Lung function measured included the ratio of maximum tidal inspiratory flow to maximum tidal expiratory flow (MTIF/MTEF) and the time to peak tidal expiratory flow to total expiratory time ratio, tPTEF/tE. Multivariable logistic regression assessed associations, and generalised structural equation modelling was used for mediation analyses.

**Results:**

The study included 1203 term infants with 89 cases of bronchiolitis hospitalisation. Tidal breathing lung function ratios were associated with bronchiolitis hospitalisations: MTIF/MTEF adjusted ORs (aORs) 2.90, 95% CI 1.20 to 7.02, p=0.018; asthma in childhood: tPTEF/tE aOR 0.97, 95% CI 0.94 to 0.99, p=0.013 and MTIF/MTEF aOR 2.94, 95% CI 1.07 to 8.02, p=0.036. MTIF/MTEF ratio mediated 11% of the total effect of asthma during pregnancy on bronchiolitis risk, and MTIF/MTEF and tPTEF/tE ratios mediated 3% of the effect of asthma during pregnancy on childhood asthma risk.

**Conclusion:**

Postnatal lung function was associated with subsequent bronchiolitis and childhood asthma. Additionally, the effect of maternal asthma during pregnancy on the outcomes was partially mediated by impaired lung function.

**WHAT IS ALREADY KNOWN ON THIS TOPIC**

- Asthma during pregnancy is one of the strongest risk factors for infant bronchiolitis and childhood asthma in offspring, but the underpinning physiological mechanisms are poorly understood.

**WHAT THIS STUDY ADDS**

- Through the leverage of two prospective birth cohorts, this study captures the effects of lung function and the risk for bronchiolitis in the first year of life and asthma at 6 years. We also show that infant lung function at 6 weeks of age may act as a moderate mediator between the association of asthma during pregnancy and infant bronchiolitis and childhood asthma.

**HOW THIS STUDY MIGHT AFFECT RESEARCH, PRACTICE OR POLICY**

- Knowing the mediator effect of lung function highlights the need for future research to explore the potential for interventions that could mitigate these effects, possibly by targeting maternal asthma management or modifying prenatal environmental exposures that influence lung development.

## Introduction

 Bronchiolitis is the most common lower respiratory tract infection and cause of hospitalisation in infancy, with an incidence ranging between 18% and 32% in the first year of life.^1^ Severe bronchiolitis in infancy, typically defined by the need for hospital admission, is associated with an increased risk of the development of childhood asthma and reduced lung function later in life.^2^,^4^ Early-life lower respiratory tract infections have also been linked to long-term respiratory outcomes. In a UK study, self-reported respiratory infections during early childhood were associated with a two-fold increased risk of premature adult death from respiratory disease, accounting for around one-fifth of such deaths.^5^

Recent evidence increasingly supports a causal relationship between early-life bronchiolitis and its negative effect on lung development.^6^,^9^ Specifically, studies have investigated whether lower infant lung function is evident prior to a bronchiolitis hospitalisation versus being merely a sequela of lower respiratory tract infection in early life.^10 11^ These studies have shown that diminished lung function may predispose to bronchiolitis in infancy. Although infant lung function testing has been employed to investigate its association with early-life lower respiratory illness, its ability to predict respiratory outcomes remains limited.^12^

Asthma during pregnancy, both controlled and uncontrolled, can lead to pregnancy complications^13^,^17^ and is a common risk factor for bronchiolitis hospitalisation.^18 19^ Infants of asthmatic mothers are at increased risk of severe bronchiolitis infection, whereas improved asthma management has been linked to a lower incidence of bronchiolitis in offspring.^14^ Notably, we have demonstrated reduced lung function in male infants born to mothers with asthma during pregnancy compared with those born to non-asthmatic mothers.^17^ Asthma during pregnancy is also a known risk factor for asthma development in childhood.^20^ It has been hypothesised that the effects of maternal asthma on child health are not solely explained by genetic factors:^21^ preclinical evidence suggests there are potential effects of in-utero exposure to maternal asthma.^22^ However, mechanisms underlying these processes remain unclear.

Given the important yet complex relationships between asthma during pregnancy, risk of bronchiolitis and asthma susceptibility in offspring,^6^,^8^ as well as the association of respiratory conditions with reduced lung function in later life,^5^ we hypothesise that these relationships may be mediated by impaired infant lung function early in life. Therefore, in an analysis of two aligned prospective birth cohorts including 1203 infants—the Australian Breathing for Life Trial (BLT) and the Swiss Basel-Bern Infant Lung Development (BILD)—we aimed to determine the association of infant lung function at 4–6 weeks of age (measured by tidal breathing flow-volume tests) and subsequent bronchiolitis hospitalisations in infancy or childhood asthma (first aim). Furthermore, we investigated whether impaired lung function in early life serves as a mediating factor in the relationship between maternal asthma during pregnancy and subsequent bronchiolitis or childhood asthma (second aim).

## Methods

The current study combines data from two multicentre prospective birth cohorts. The BLT birth cohort includes only infants born to women with mild to moderate asthma during pregnancy who participated in a randomised intervention for asthma management during pregnancy.^23^ Asthma during pregnancy was defined as self-reported, doctor-diagnosed asthma with either current asthma symptoms or the use of inhaled asthma medication. The BILD birth cohort includes infants born to mothers with asthma and non-asthmatics.^24^ A history of maternal asthma was defined as self-reported, doctor-diagnosed asthma at any time, along with a specific question on asthma during pregnancy.

Both studies have aligned methodologies, and a combined data analysis has been described previously.^17^ Infants were followed up at 4–6 weeks of age and again at 5–6 years. For the current analyses, infants were excluded if they had been hospitalised for bronchiolitis before lung function testing or had a respiratory illness in the 2 weeks prior to testing. Preterm infants were included (details in online supplemental material). Written informed parental consent was obtained at the time of enrolment. Both studies are approved by the local human ethics committees.

### Lung function

Both cohorts used identical equipment (details in online supplemental material). Briefly, testing was conducted during behaviourally defined quiet natural sleep using an infant mask (sizes 0, 0/1 and 1; Homedica, Huenenberg, Switzerland), following European Respiratory Society/American Thoracic Society standards for infant lung function testing.^25 26^

The analysis examined respiratory rate, mean tidal volume, minute ventilation and the ratio of time to reach peak tidal expiratory flow (PTEF) as a percentage of total expiratory time (tPTEF/tE). In addition, to compare differences between inspiratory and expiratory time and flow, we calculated the ratio of the maximum inspiratory flow to maximum expiratory flow (MTIF/MTEF), the ratio of time to peak tidal inspiratory flow to peak tidal expiratory flow (PTIF/PTEF) and the ratio of inspiratory time to expiratory time (tI/tE). Higher ratios indicate shorter inspiratory times, which may limit the time available for full alveolar filling and equilibration before the onset of expiration; this could contribute to less effective ventilation by reducing tidal volume or altering ventilation distribution.

### Bronchiolitis in infancy

In both studies, bronchiolitis hospitalisations (encompassing both emergency department presentations and admissions) were identified through electronic medical records during the first year of life. To ensure comparability between the two cohorts, the bronchiolitis hospitalisation criteria were standardised (details in online supplemental material).

### Asthma in childhood

Follow-up assessments for children in the BLT and BILD studies were conducted at 5–6 years of age to determine asthma diagnosis. Parents were interviewed to confirm whether a doctor had diagnosed their child with asthma, subsequently verified by a paediatric pulmonologist who reviewed the clinical information provided during the interview (details in online supplemental material).^27^,^29^

### Patient and public involvement

It was not appropriate or possible to involve patients or the public in the design, conduct, reporting or dissemination plans of our research. In the BLT, mothers were recruited as participants in the maternal randomised controlled trial, and their infants were enrolled for follow-up, including lung function assessments during infancy. In the BILD cohort, parents were recruited in the antenatal clinic, and their infants attended scheduled visits where lung function testing and other assessments were performed. Results were shared with families through individual summary reports, newsletters and on the study websites.

### Statistical analyses

To investigate whether infant lung function influences the relationship between risk factors and bronchiolitis (first aim, part one) or asthma in childhood (first aim, part two), we performed multivariable logistic regression, adjusting for a priori defined confounders.^17 30 31^ The final models included maternal asthma during pregnancy, sex, prematurity, having siblings at birth, weight at study date (z-score), season of birth, mode of delivery (vaginal or caesarean), breastfeeding at the time of lung function assessment and study centre. Sensitivity analysis incorporated maternal atopy (defined as allergic rhinitis or eczema in addition to an asthma diagnosis), and we tested the robustness of the model by, in another model, excluding infants whose mother smoked during pregnancy.

To address the study’s second aim, we conducted mediation analyses using generalised structural equation modelling,^32^ with the binomial family and logit link function in Stata, which appropriately addressed the binary outcomes (bronchiolitis and asthma), enabling accurate probability. The total effect (TE) was decomposed into direct effect (DE, not mediated by infant lung function) and indirect effect (IE, mediated through infant lung function), with the mediation contribution quantified to determine the effect size of the indirect pathway through infant lung function, following the method described in Mehmetoglu.^33^ This decomposition allowed us to assess whether lung function served as a potential mechanistic pathway—beyond purely genetic inheritance—linking maternal asthma to child respiratory outcomes. To evaluate the effect size of an IE, the ratio of the IE to the DE is calculated (RID). We used the formula RID=a*b/c as described by Mehmetoglu.^33^ Results are presented as adjusted β coefficients for infant lung function, exponentiated to show the adjusted OR for the outcomes.Online supplemental Figure S1 provides a visual summary of the mediation framework.

A p value (p)<0.05 was considered statistically significant (details in online supplemental material). All analyses were performed using Stata 16.1 (StataCorp, College Station, Texas, USA).

## Results

### Baseline characteristics

A total of 1203 infants (BLT n=383, all born to mothers with asthma; BILD n=820, n=778 born to non-asthmatics and n=42 born to mothers with asthma) were included in the analysis (online supplemental Figure S2). Of these, 89 (7.1%) were hospitalised for bronchiolitis during their first year of life. Hospitalised infants were more likely than non-hospitalised infants to have been born to a mother with asthma during pregnancy (48.3% vs 34.3%), have siblings at birth (62.9% vs 44.4%), be male (61.8% vs 50.8%), have had mild respiratory symptoms in the first 2 weeks of life (15.7% vs 5.7%), have a history of preterm birth (25.8% vs 13.8%) and have a lower rate of breastfeeding (79.8% vs 89.1%) (table 1 and online supplemental table S1).

**Table 1:** Baseline characteristics of infants stratified by hospitalisation status for bronchiolitis

	Non-hospitalisation (n=1114)	Hospitalisation (n=89)	P value‡
Maternal baseline characteristics			
Maternal asthma during pregnancy *n (%)*	382 (34.3)	43 (48.3)	**0.006**
Maternal smoking during pregnancy *n (%)*	76 (6.8)	7 (7.9)	0.709
Infant baseline characteristics			
Having siblings at birth *n (%)*	495 (44.4)	56 (62.9)	**0.001**
Prematurity *n (%)*	154 (13.8)	23 (25.8)	**0.002**
Male *n (%)*	566 (50.8)	55 (61.8)	**0.046**
Delivery type:			0.062
Vaginal *n (%)*	770 (69.1)	53 (59.5)	
C-section *n (%)*	344 (30.9)	36 (40.5)	
Season of birth:			0.194
Winter *n (%)*	213 (19.1)	20 (22.5)	
Spring *n (%)*	354 (31.8)	21 (23.6)	
Summer *n (%)*	286 (25.7)	22 (24.7)	
Autumn *n (%)*	261 (23.4)	26 (29.2)	
Gestational age in weeks*	39.4 (2.0)	38.9 (3.1)	**0.008**
Birth weight in kg†	3.2 (0.7)	3.2 (0.8)	0.677
Birth length in cm†	49.2 (3.6)	49.1 (4.3)	0.744
Infant characteristics at test			
Symptoms at first 2 weeks of life *(%)*	63 (5.7)	14 (15.7)	**0.001**
Breastfeeding at test date *n (%)*	990 (89.1)	71 (79.8)	**0.008**
Postmenstrual age at test in weeks†	45.2 (1.6)	45.0 (1.7)	0.255
Weight at test in kg†	4.5 (0.6)	4.7 (0.7)	0.013
Length at test in cm†	55.0 (2.8)	55.4 (3.0)	0.212

P values <0.05 are shown in bold.

* Values show median (IQR*)*.

† Values show mean (SD).

‡ Comparing all infants who were hospitalised with bronchiolitis during their first year of life to all infants who were not hospitalised. Groups were compared using either a t-test or a χ2 test as appropriate.

### Infant lung function and bronchiolitis hospitalisation (first aim, part one)

Univariable analyses are presented in online supplemental table S2. Representative tidal breathing flow curves are shown in online supplemental Figure S3, and correlations between all tidal breathing parameters are provided in online supplemental Figure S4.

Multivariable regression analysis revealed significant associations between higher MTIF/MTEF (adjusted OR (aOR) 2.90; 95% CI 1.20 to 7.02, p=0.018) and PTIF/PTEF (aOR 2.50; 95% CI 1.02 to 6.12, p=0.046) with increased odds of bronchiolitis hospitalisations. Conversely, higher tI/tE ratios (aOR 0.17; 95% CI 0.03 to 0.87, p=0.033; table 2) were negatively associated with bronchiolitis hospitalisations. No associations were found for tPTEF/tE (tables2  3).

**Table 2:** Multivariable logistic regression analysis bronchiolitis OR and asthma in childhood risk per unit increase in lung function parameters

	Bronchiolitis		Asthma	
	Combined (n=1114 not hospitalised, n=89 bronchiolitis hospitalised		Combined (n=694 total, n=100 asthma)	
	aOR (95% CI)	P value	aOR (95% CI)	P value
Volumes and respiratory rate				
TV, mL	0.97 (0.80 to 1.17)	0.727	1.03 (0.85 to 1.24)	0.785
Minute ventilation, min/mL	1.00 (1.00 to 1.00)	0.647	1.00 (0.99 to 1.00)	0.472
RR, min	1.00 (0.98 to 1.02)	0.917	0.99 (0.97 to 1.02)	0.870
Ratios				
tPTEF/tE, %	0.99 (0.97 to 1.02)	0.586	**0.97 (0.94 to 0.99**)	**0.013**
MTIF/MTEF	**2.90 (1.20 to 7.02**)	**0.018**	**2.94 (1.07 to 8.02**)	**0.036**
PTIF/PTEF	**2.50 (1.02 to 6.12**)	**0.046**	**3.00 (1.11 to 8.15**)	**0.031**
tI/tE	**0.17 (0.03 to 0.87**)	**0.033**	0.18 (0.03 to 1.19)	0.072

Multivariable analysis adjusted for hospital admission due to bronchiolitis, male sex, prematurity, having siblings at birth, caesarean section, z-score for weight at test, season of birth as a categorical variable, breastfeeding at the time of lung function testing and study centre. P values <0.05 are shown in bold.

Asthma models were adjusted for hospital admission or ED presentation due to bronchiolitis, maternal asthma during pregnancy, male sex, current breastfeeding at the time of lung function testing and age at test.

aOR, adjusted OR; ED, emergency department; MTIF/MTEF, ratio of medium-term inspiratory flow to medium-term expiration flow; PTIF/PTEF, ratio of time to peak tidal inspiratory flow to peak tidal expiratory flow; RR, respiratory rate; tI/tE, ratio of inspiratory time to expiratory time; tPTEF/tE, time to peak tidal expiratory flow divided by total expiratory time; TV, tidal volume.

**Table 3:** Baseline characteristics of infants stratified by asthma in childhood status

	Non-asthma (n=594)	Asthma (n=100)	P value
Maternal baseline characteristics			
Maternal smoking during pregnancy *n (%)*	35 (5.9)	9 (8.8)	0.257
Maternal asthma during pregnancy *n (%)*	125 (21.0)	83 (83.0)	**<0.001**
Infant baseline characteristics			
Having siblings at birth *n (%)*	282 (47.3)	47 (46.1)	0.817
Prematurity *n (%)*	77 (12.9)	13 (12.8)	0.961
Male *n (%)*	290 (48.7)	65 (63.7)	**0.005**
Delivery type:			0.882
Vaginal *n (%)*	425 (71.3)	72 (70.6)	
C-section *n (%)*	171 (28.7)	30 (29.4)	
Season of birth:			**0.046**
Winter *n (%)*	109 (18.3)	25 (24.5)	
Spring *n (%)*	191 (32.1)	34 (33.3)	
Summer *n (%)*	146 (24.5)	30 (29.4)	
Autumn *n (%)*	150 (25.2)	13 (12.8)	
Gestational age in weeks*	39.6 (2.0)	39.1 (2.0)	0.835
Birth weight in kg†	3.2 (0.7)	3.4 (0.6)	**0.010**
Birth length in cm†	48.9 (3.4)	51.0 (3.3)	**<0.001**
Infant characteristics at test			
Breastfeeding at test date *n (%)*	562 (94.5)	74 (72.6)	**<0.001**
Symptoms at first 2 weeks of life (*%)*	30 (5.0)	15 (14.7)	**<0.001**
Postmenstrual age at test in weeks†	45.0 (1.4)	45.6 (1.9)	**0.003**
Weight at test in kg†	4.5 (0.6)	4.8 (0.7)	**<0.001**
Length at test in cm†	54.8 (2.4)	55.7 (2.4)	**0.003**
Bronchiolitis in the first year of life	30 (5.0)	14 (13.7)	**0.001**

P values <0.05 are shown in bold.

Groups were compared using either a t-test or a χ2, as appropriate.

* Values are presented as median (IQR)*. *

† Values are presented as mean (SD)*.*

To assess the robustness of the results, a sensitivity analysis excluding infants whose mothers reported smoking during pregnancy revealed similar results for MTIF/MTEF (aOR 3.20; 95% CI 1.28 to 0.00, p=0.013) and tI/tE (aOR 0.17; 95% CI 0.03 to 1.16, p=0.040). In another sensitivity analysis, the results also remained consistent after excluding preterm infants (aOR 3.20; 95% CI 1.28 to 8.00, p=0.013). Additionally, no associations were found between maternal atopy and the odds of having bronchiolitis (aOR 1.12; 95% CI 0.71 to 2.01, p=0.629).

### Infant lung function and childhood asthma (first aim, part two)

We followed up 694 children at 5–6 years of age (BLT n=184; BILD n=510). Among these, 14.4% (n=100) had doctor-diagnosed asthma. Table 3 gives baseline characteristics of asthmatic and non-asthmatic children. Online supplemental table S4 gives univariable analyses of lung function parameters.

Multivariable regression analysis revealed that higher tPTEF/tE was associated with a lower odds of childhood asthma (aOR 0.97; 95% CI 0.94 to 0.99 p=0.013). In contrast, higher MTIF/MTEF (aOR 2.94; 95% CI 1.07 to 8.02, p=0.036) and PTIF/PTEF ratios (aOR 3.00; 95% CI 1.11 to 8.15, p=0.031) were associated with increased odds of childhood asthma (tables2  3).

### Bronchiolitis hospitalisation is mediated by infant lung function (second aim, part one)

**Figure 1:**
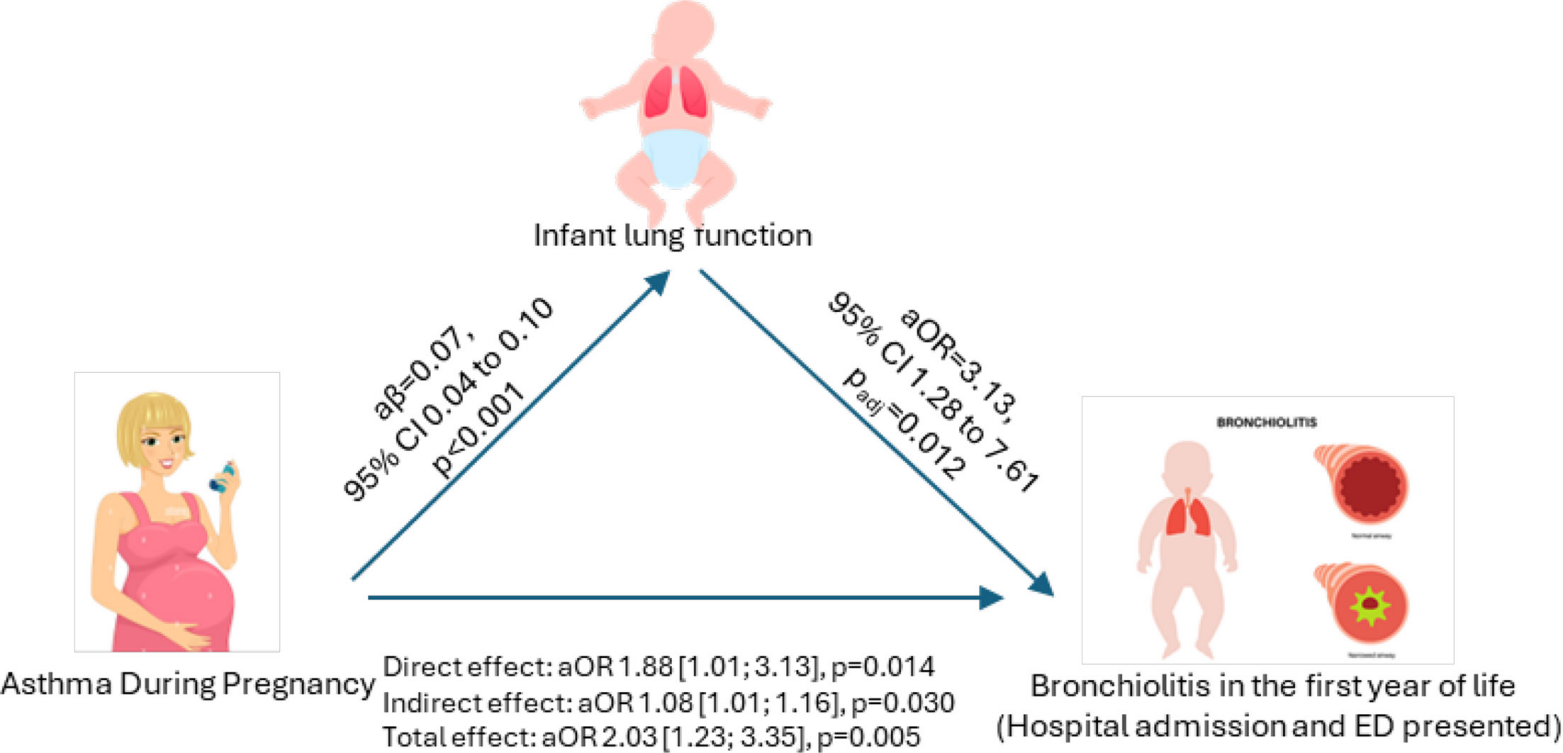
Structural equation modelling of the mediation effect of the MTIF/MTEF ratio on the relationship between asthma during pregnancy and bronchiolitis hospitalisation in the first year of life. The model is adjusted for male sex, prematurity, having siblings at birth, caesarean section, z-score for weight at the time of testing, season of birth, current breastfeeding at the time of lung function testing and age at testing. aOR, adjusted OR; ED, emergency department. MTIF/MTEF, ratio of medium-term inspiratory flow to medium-term expiration flow.

We considered infant lung function (MTIF/MTEF) as an IE that may mediate the relationship between asthma during pregnancy and the risk of bronchiolitis hospitalisation in the first year of life (online supplemental Figure S1). Maternal asthma during pregnancy significantly increased the odds of bronchiolitis through a DE (aOR 1.88; 95% CI 1.01 to 3.13, p=0.014). Additionally, asthma during pregnancy was associated with higher MTIF/MTEF (aβ 0.07; 95% CI 0.04 to 0.10, p<0.001), and a higher MTIF/MTEF was, in turn, associated with an increased odds of bronchiolitis (aOR 3.13; 95% CI 1.28 to 7.61, p=0.012). The IE by the MTIF/MTEF ratio was significant (aOR 1.08; 95% CI 1.01 to 1.16, p=0.030, figure 1), suggesting that a one-unit increase in MTIF/MTEF due to asthma during pregnancy led to an 8.2% increase in the odds of bronchiolitis (95% CI 1.7 to 27.9%) when the RID was calculated. Overall, MTIF/MTEF mediated 11.1% (95% CI 1.8 to 27.0%) of the association between maternal asthma during pregnancy and infant bronchiolitis.

### Childhood asthma is partially mediated by infant lung function (second aim, part two)

Next, we investigated whether tPTEF/tE and MTIF/MTEF ratios mediate the effect of asthma during pregnancy on childhood asthma risk. In our mediation analysis, maternal asthma during pregnancy was significantly associated with childhood asthma (DE aOR 18.91; 95% CI 10.18 to 34.81, p<0.001). Asthma during pregnancy was also associated with a lower tPTEF/tE (aβ −3.67; 95% CI −4.99 to −2.36, p<0.001). In turn, a higher tPTEF/tE was associated with lower odds of childhood asthma (aOR 0.96; 95% CI 0.94 to 0.96, p=0.014).

**Figure 2:**
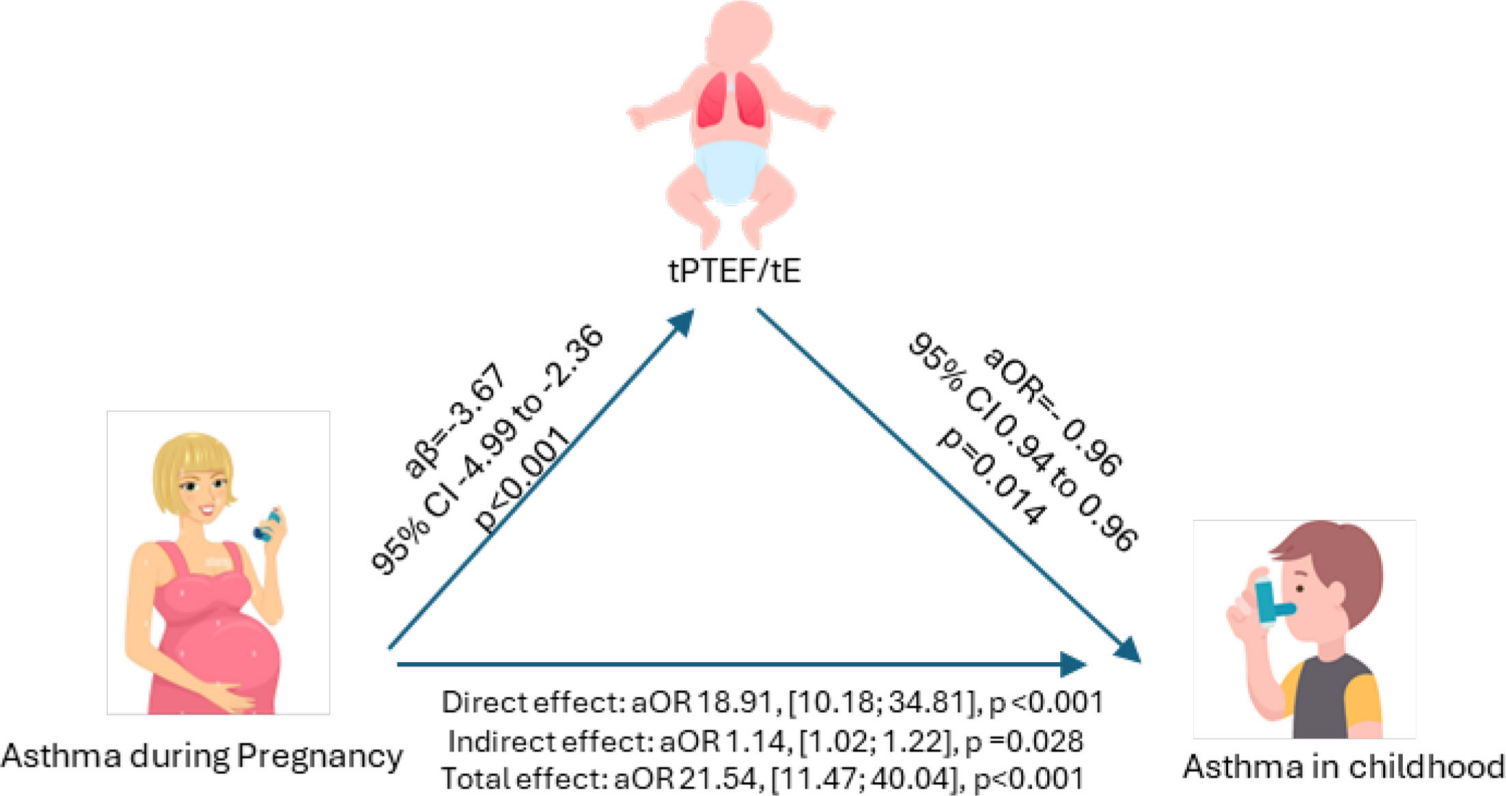
Structural equation modelling of the mediation effect of the tPTEF/tE ratio on the relationship between asthma during pregnancy and asthma in childhood. The model is adjusted for male sex, prematurity, having siblings at birth, caesarean section, z-score for weight at the time of testing, season of birth, current breastfeeding at the time of lung function testing and age at testing. aOR, adjusted OR; tPTEF/tE, time to peak tidal expiratory flow divided by total expiratory time.

The IE of maternal asthma during pregnancy on childhood asthma, mediated through tPTEF/tE, was significant (aOR 1.14; 95% CI 1.02 to 1.22, p=0.028; figure 2). This suggests that a decrease in tPTEF/tE due to maternal asthma during pregnancy led to a 14% increased odds of childhood asthma (95% CI 1.7% to 27.9%). tPTEF/tE mediated 4.3% (95% CI 1.7% to 7.8%) of the association between maternal asthma and childhood asthma.

We found that the association between maternal asthma during pregnancy and childhood asthma was mediated by MTIF/MTEF, with a significant TE (aOR 19.86; 95% CI 10.80 to 36.97, p < 0.001), DE (aOR 18.54; 95% CI 9.97 to 34.12, p < 0.001) and IE (aOR 1.08; 95% CI 1.01 to 1.17, p=0.048). Additionally, increased MTIF/MTEF was associated with a higher odds of childhood asthma (aOR 1.15; 95% CI 1.02 to 34.12, p=0.025) and mediated approximately 3% (95% CI 2.1% to 7.0%) of this effect.

## Discussion

In this analysis from two birth cohort studies, we demonstrate that maternal asthma during pregnancy and impaired tidal breathing lung function shortly after birth are associated with bronchiolitis hospitalisations in the first year of life and asthma development in early childhood. While both risk factors have independent effects, a novel aspect of our study is that reduced infant lung function moderately mediated the association between asthma during pregnancy and both infant bronchiolitis and childhood asthma.

Previous studies have established an association between maternal asthma during pregnancy and infant bronchiolitis and that better asthma management may reduce adverse offspring outcomes in infancy.^14 16 34 35^ Our study corroborates prior research showing that infants who develop later lower respiratory tract infections exhibit elevated airway resistance^36 37^ and reduced lung function.^38^ We further suggest that these associations might be mediated by adverse effects on lung function impairment, which may track into adulthood, predisposing individuals to early-onset chronic obstructive pulmonary disease and premature death.^39^,^43^

Several aspects of our findings have clinical relevance. Abnormal tidal breathing flow volume parameters, including higher PTIF and prolonged tE, have been observed in infants with acute viral bronchiolitis admitted to the intensive care unit (ICU) and may guide high-flow nasal cannula therapy.^44^ Of note, we found similar qualitative differences in tidal breathing patterns in healthy infants who were hospitalised for bronchiolitis later in life, although quantitatively less severe. This suggests that abnormal tidal breathing may precede severe bronchiolitis. Additionally, increased MTIF/MTEF ratios, reflecting relatively shorter inspiratory times, were associated with bronchiolitis and childhood asthma, potentially indicating altered breathing patterns that may affect ventilation efficiency or airway dynamics in early life. However, unlike our previous findings linking lower tPTEF/tE in male offspring of asthmatic mothers, our current results did not indicate that tPTEF/tE has a mediating effect on bronchiolitis. Instead, expiratory airflow limitation, reflected in a higher MTIF/MTEF ratio, may be a more sensitive measure for the risk of bronchiolitis.

Conversely, lower tPTEF/tE ratios in infancy partially mediated the risk of childhood asthma. Lower tPTEF/tE ratios in infancy have been associated with structural airway abnormalities on high-resolution CT, including smaller ratios for inner and outer diameter ratios, total area, luminal area and wall area by age 26.^45^ We hypothesise that the early signs of obstruction observed in a lower tPTEF/tE may have long-term implications for the development of childhood asthma. second, our results further highlight the need for early-life strategies to promote healthy foetal and neonatal lung growth, particularly in the offspring of mothers with asthma during pregnancy, who have an increased risk of respiratory disease later in life.

The precise mechanisms whereby adverse effects at the foetal–maternal interface disturb lung growth are complex and remain incompletely understood. For instance, in-utero smoke exposure may impact lung development through multiple pathways.^46^ However, our findings remained consistent even after conducting a sensitivity analysis excluding infants whose mothers smoked during pregnancy, indicating that smoking was not responsible for the observed effects.

Experimental studies suggest that allergic airway disease in pregnancy may promote foetal eosinophilia^47^ and that maternal allergen exposure in pregnancy can alter the typical Th2 immune phenotype of the offspring.^47^ In a further sensitivity analysis controlling for maternal atopy, no associations were found with atopy. Previous analysis from the BLT cohort revealed elevated eosinophil and reduced neutrophil levels in the cord blood of infants who were later hospitalised with bronchiolitis.^48 49^ Together, these findings suggest that maternal asthma during pregnancy may have a stronger association with offspring lung function and respiratory health than maternal atopy. By examining the DE and IE, our mediation analysis might help to verify whether maternal asthma is associated with infant outcomes via its impact on lung function. Our rationale for including lung function as a mediator is based on previous evidence of how early-life lung function may be reflected in utero and in early postnatal airway development, which might be influenced by maternal asthma.^50^ This suggests that early lung function impairment may be a key step in the causal pathway linking maternal asthma to these outcomes.

Strengths of our analysis include a large sample size, prospective follow-up and aligned methodology. Combining data from both cohorts allowed us to uniquely examine early-life lung function as a mediator in the relationship between maternal asthma during pregnancy and adverse health outcomes in offspring. Additionally, although the BLT cohort consists of asthmatic mothers, all asthma cases, similar to those in the BILD cohort, are of mild to moderate severity.^23 24^

Our analysis has several limitations. Although the asthma cases are of mild to moderate severity, we do not have the power to fully address asthma exacerbations during pregnancy or medication use. Additionally, combining data from two geographically distant birth cohorts raises the possibility of uncontrolled genetic, environmental and study-specific effects that could potentially confound our results. Even though we have a large sample size, we need to acknowledge that the number of cases is relatively small, and further research is warranted to confirm and further explore our findings. Another limitation is that we could not adjust for the length of stay or the type of virus in the children in the bronchiolitis group. Our analysis may also be affected by a selection bias, as the BLT cohort only included offspring born to mothers with asthma during pregnancy. Nevertheless, the study methodologies were optimally aligned, particularly the measurement of the primary outcomes and infant lung function, as well as adjustments to account for factors such as weight at test, gestational age and study centre. Mediation analysis alone cannot distinguish whether this IE is due to genetic inheritance, foetal programming or postnatal environmental influences on lung development. Nevertheless, results from the mediation analysis are consistent with the hypothesis that the maternal asthma activity creates an intrauterine environment that may impair lung functional development, potentially leading to subsequent vulnerability for respiratory morbidity.

In conclusion, impaired tidal breathing lung function shortly after birth is associated with bronchiolitis hospitalisation and asthma development in childhood and partially mediates the association between asthma during pregnancy and these adverse respiratory outcomes in offspring. Although the offspring of asthmatic mothers may be clinically asymptomatic at birth, the preconditions for later respiratory chronic disease may already be programmed in utero.^50^ Consequently, this may imply that the prevention of chronic disease in early childhood may begin during pregnancy. Future studies are warranted to determine whether improving asthma control in pregnant women (through maternal medication use) can reduce the prevalence of bronchiolitis and asthma in their offspring by improving lung function. We further propose that exploring these hypotheses is of relevance to general paediatrics, as they may serve as a model for other complex paediatric chronic diseases influenced by genetic and environmental determinants.

## Supplementary material

10.1136/bmjresp-2025-003679 online supplemental file 1

## Data Availability

Data are available upon reasonable request.
