# Supplementary material for: Early-life lung function deficits partially explain the link between maternal asthma and bronchiolitis or asthma in offspring
Source: BMJ Open Respir Res. 2026 May 18;13(1):e003679. doi: 10.1136/bmjresp-2025-003679 (PMC13185018; doi:10.1136/bmjresp-2025-003679)
Supplement: online supplemental file 1 [file bmjresp-13-1-s001.pdf]

Supplementary Material

Early-life lung function deficits partially explain the link between maternal asthma and bronchiolitis or asthma in offspring

Carla Rebeca Da Silva Sena[1,2,3], Adam Collison[2], Vanessa E Murphy[2,4], Gabriela Martins Costa Gomes[2], Olga Gorlanova[1,3], Noëmi Künstle[1,3], Céline Rüttimann[1,3], Sven Schulzke[1], Benjamin Stoecklin[1], Jakob Usemann[1], Ruth Steinberg[,3], Sophie Yammine[3], Paul D Robinson[5,6,7], Peter D Sly[6], Philipp Latzin[1,3], Peter G Gibson[2,4,8], Urs Frey[1#], Joerg Mattes[2,9,#].

[1]University Children's Hospital Basel UKBB, University of Basel, Basel, Switzerland

[2]Hunter Medical Research Institute and Asthma & Breathing research program, The University of Newcastle, Newcastle, New South Wales, Australia

[3]Department of Paediatrics, Inselspital University Hospital Bern, Division of Paediatric Respiratory Medicine and Allergology, Bern, Switzerland

[4]School of Medicine and Public Health, College of Health, Medicine and Wellbeing, The University of Newcastle, Newcastle, New South Wales, Australia

[5]Department of Respiratory Medicine, The Children's Hospital at Westmead, Sydney, New South Wales, Australia

[6] South Brisbane, Children's Health and Environment Program, Child Health Research Centre, The University of Queensland, Brisbane, Queensland, Australia

[7] Discipline of Paediatrics and Child Health, University of Sydney, Sydney, New South Wales, Australia

[8]Respiratory & Sleep Medicine Department Newcastle, John Hunter Hospital, New South Wales, Australia

[9]Paediatric Respiratory & Sleep Medicine Department, John Hunter Children's Hospital, Newcastle, New South Wales, Australia

#Correspondence:

Prof Joerg Mattes and Prof Urs Frey

E-mail: joerg.mattes@newcastle.edu.au; Address: Hunter Medical Research Institute, Lookout Road, 2305 New Lambton, Australia

E-mail: urs.frey@ukbb.ch; Address: University Children's Hospital Basel, Spitalstrasse 33, 4056 Basel, Switzerland

Authors' contributions: JM, UF and AC conceived the project and supervised analysis; CRDSS, AC and GMCG conducted analyses and drafted data tables; CRDSS, GMCG, PDGB, NK, OG and CR analysed the quality of lung function data; PGG, VEM and JM conducted the Breathing for Life Trial (BLT) pregnancy study; JM, PGG, VEM, AC, PDR, PDS, CRDSS and EQA conducted BLT infant and childhood age follow-up; UF, PL, NK, OG, CR, SS, RS, SY and JU conducted BILD infant and childhood follow-up; JM supervised the BLT infant follow-up; UF and PL supervised BILD study follow-up; CRDSS and JM wrote a draft manuscript and figures; all authors edited the final version of the manuscript. UF is the guarantor.

Methods

The BLT birth cohort (https://www.breathingforlife.com.au) includes offspring of mothers with mild to moderate asthma during pregnancy, who were randomised into two asthma management groups [1]. No difference in perinatal outcomes was observed between asthma management guided by exhaled nitric oxide levels and usual care [2]. Pregnant women were recruited from six Australian public hospital antenatal clinics (7 March 2013 to 11 June 2019). Participants were required to have doctor-diagnosed asthma, symptoms of asthma and/or asthma medication use (prior 12 months), were aged ≥18 years, and between 12 and 23 completed weeks of gestation at randomization. Following consent, infants underwent lung function testing at six weeks of corrected age [3], with assessments conducted in Newcastle and Sydney from May 2014 to December 2019.

The prospective BILD cohort (https://www.bild-cohort.ch) comprises neonates recruited since 1999 in the region of Bern and since 2012 in Basel, Switzerland [4, 5]. Infants were followed up at 4–6 weeks of corrected age and underwent lung function testing included in the current analysis from 1999 to September 2022. Additionally, in the first year of life, infants were monitored weekly via phone calls. Maternal asthma history was defined as self-reported, doctor-diagnosed asthma in a questionnaire or during the interview. Maternal asthma during pregnancy was classified as mild to moderate severity. Potential risk factors were assessed through interviews at the lung function measurement visit and during the weekly phone calls using standardised questionnaires. At the age of 5–6 years children were invited to the study clinic for the follow-up visit that included lung function testing and a questionnaire to ascertain if the child had doctor-diagnosed asthma.

The Australian BLT cohort was approved by the Hunter New England Local Health District Human Research Ethics Committee (2019/ETH03856, 2020/ETH02881). The Swiss BILD

cohort was approved by the Ethics Committee of Northwestern and Central Switzerland (2022–00336) and the Bernese Cantonal Ethics Research Committee (2019-01072).

Lung function

Both cohorts used identical equipment which tests infants during behaviourally defined quiet natural sleep, using an infant mask (sizes 0, 0/1 and 1; Homedica, Huenenberg, Switzerland), according to the European Respiratory Society/American Thoracic Society (ERS/ATS) standards of infant lung function testing [6, 7]. Mask size dead space was corrected during analysis. Flow was measured using an ultrasonic flow meter in both cohorts (Spiroson; Eco Medics, Duernten, Switzerland). Testing protocols between sites only differed in the duration of testing, as described by Belinelo et al. [3].

Bronchiolitis in infancy (first aim, part one)

The history of hospitalisation for bronchiolitis was collected from hospital medical records. In the BLT study, bronchiolitis hospitalisations—encompassing both presentations and admissions—were identified through electronic medical records within the New South Wales (NSW) Health district networks during the first year of life.

In the BILD study, respiratory symptoms, including wheezing and lower respiratory tract symptoms, were assessed weekly using a standardised scoring system that categorizes symptoms by severity during the first year of life [8]. In the current analysis, extensive verification of weekly phone call notes was conducted to identify infants with a history of bronchiolitis. Subsequently, medical records were assessed to ascertain infants admitted or presented to the emergency department (ED) with a clinical diagnosis of bronchiolitis.

Asthma in childhood (first aim, part two)

At 5–6 years of age, follow-up assessments for children participating in both the BLT and BILD studies were conducted, including a visit to perform lung function testing and to

ascertain an asthma diagnosis. During this visit, parents were interviewed to report whether their child had been diagnosed with asthma by a doctor. Additionally, standardised International Study of Asthma and Allergies in Childhood (ISAAC) questionnaires were used to assess respiratory symptoms and medication use in the last 12 months [9-11].

For the BLT cohort, the specific question asked during the interview with the parent/guardian was: “Has your child been diagnosed with asthma by a doctor?”. If the answer was yes, a follow-up question would be asked: “How old was your child when they received the asthma diagnosis?”. For the BILD cohort, the question asked during the interview with the parent/guardian was: “Have you ever been told by a doctor that your child has asthma?”.

Statistical analyses

Summary statistics of study subjects’ characteristics were reported in terms of frequency and percentage when categorical variables, and in terms of mean and standard deviations (SD) or median and interquartile range (IQR=first quartile–third quartile) as appropriate when continuous variables. Comparisons between groups were tested using a t-test if approximately normally distributed, and a Wilcoxon test if non-normally distributed. A p-value (p) <0.05 was considered significant.

To investigate the risk factors for bronchiolitis according to lung function, we performed multivariable logistic regression adjusting for a priori confounders [3, 12, 13]. Our final model included confounders such as sex, prematurity, being a firstborn, weight at the study date (transformed to a z-score), season of birth as categories, mode of delivery (vaginal birth or Caesarean section), and breastfeeding at the time of lung function assessment. Additionally, asthma during pregnancy was included to ascertain its effect estimate. For the sensitivity analyses, maternal atopy was incorporated into the model. We tested the robustness in one model excluding preterm infants, and in another model excluding infants whose mother smoked during pregnancy.

Mediation analyses were conducted to achieve the study's second aim, examining whether the association between maternal asthma during pregnancy and the risk of bronchiolitis hospitalisation (second aim, part one) and asthma in childhood (second aim, part two) is mediated by tidal parameters. This was performed using generalised structural equation modelling (GSEM package in Stata 16.1) [14], with a binomial family and logit link function to appropriately address the binary outcome, enabling accurate probability estimation of the outcomes. The Total Effect (TE) of maternal asthma during pregnancy on bronchiolitis hospitalisation was decomposed into Direct Effect (DE) and Indirect Effect (IE), with the proportion of mediation contribution quantified to determine the effect size of the indirect pathway through infant lung function, following the method described by Mehmetoglu [15]. To evaluate the effect size of an indirect effect is to take the ratio of the indirect effect to the direct effect (RID), we used the formula RID= a*b/c. Figure S1 provides a visual summary of the mediation framework.

All mediation models included adjustments for defined a priori confounders. To analyse the effect of maternal asthma on lung function, and subsequent incidence of bronchiolitis/asthma the model was further adjusted for sex, prematurity, firstborn status, weight at the study date, birth season, delivery mode, breastfeeding status at the time of lung function assessment, and age at test. All analyses were performed using STATA 16.1.

Supplementary Tables

Supplementary Table 1. Baseline characteristics in BLT and BILD study infants and combined cohorts, stratified by asthma and no asthma in childhood

	BLT cohort (n=184)		BILD cohort (n=510)		Combined (n=694)		
	**Non-asthma (n=102)**	**Asthma (n=82)**	**Non-asthma (n=492)**	**Asthma (n=18)**	**Non-asthma (n=594)**	**Asthma (n=100)**	**p-value**
**Maternal baseline characteristics**							
Maternal smoking during pregnancy *n (%)*	9 (8.8)	7 (8.5)	26 (5.3)	2 (11.1)	35 (5.9)	9 (9)	0.238
Maternal asthma during pregnancy n (%)	102 (100)	82 (100)	23/492 (4.7)	1/18 (5.6)	**125 (21.0)**	**83 (83.0)**	**<0.001**
**Infant baseline characteristics**							
Having siblings at birth *n (%)*	39 (38.2)	33 (40.2)	242 (49.2)	13 (72.2)	281 (47.3)	46 (46.0)	0.809
Prematurity *n (%)*	6 (5.9)	12 (14.6)	71 (14.4)	1 (5.6)	77 (13.0)	13 (13.0)	0.992
Male *n (%)*	46 (45.1)	50 (61.0)	243 (49.4)	13 (72.2)	**289 (48.7)**	**63 (63.0)**	**0.008**
Delivery type:							0.778
Vaginal *n (%)*	65 (63.7)	56 (68.3)	359 (73.0)	14 (77.8)	424(71.4)	70 (70.0)	
C-section *n (%)*	37 (36.3)	26 (31.7)	133 (27.1)	4 (22.2)	170 (28.6)	30 (30.0)	
Season of birth:							**0.015**
Winter *n (%)*	29 (28.4)	25 (30.5)	107 (21.8)	0 (0%)	136 (22.9)	25 (25.0)	
Spring *n (%)*	21 (20.6)	24 (29.3)	145 (29.5)	6(33.3)	166 (28.0)	30 (30.0)	
Summer *n (%)*	28 (27.5)	24 (29.3)	117 (23.8)	8 (44.4)	145 (24.4)	32 (32.0)	
Autumn *n (%)*	24 (23.5)	9 (11.0)	123 (25.0)	4 (22.2)	147 (24.8)	13 (13.0)	
Gestational age in weeks[†]	39.4 (1.7)	39.1 (2.1)	39.6 (2.1)	40.4 (2.2)	39.6 (2.0)	39.1 (2.0)	0.828
Birth weight in kg*	3.4 (0.6)	3.4 (0.6)	3.2 (0.7)	3.4 (0.7)	**3.2 (0.7)**	**3.4 (0.6)**	**0.018**
Birth length in cm*	50.8 (3.3)	51.4 (3.2)	48.6 (3.3)	49.7 (3.4)	**48.9 (3.4)**	**51.0 (3.3)**	**<0.001**
Exclusive breastfeeding until test date *n (%)*	82 (80.4)	55 (67.1)	478 (97.2)	17 (95.0)	**560(94.3)**	**72 (72.0)**	**<0.001**
Bronchiolitis in the first year of life	9 (8.8)	14 (17.1)	21 (4.3)	0 (0%)	**30 (5.1)**	**14 (14.0)**	**0.001**
Age at follow-up in months*	65.5 (12.9)	66.6 (13.1)	70.7 (4.6)	69.5 (5.4)	**69.6 (7.1)**	**67.2 (12.1)**	**0.002**

*Values are shown as *mean (SD)*/[†]Values are shown as *median (IQR)*. Comparing children with and without parent-reported asthma diagnoses within the BLT cohort, the BILD cohort and in the combined cohorts. For the combined analysis, groups were compared using either a t-test or a chi-square test as appropriate. P-values <0.05 are shown in bold. BILD, Bern Infant Lung Development; BLT, Breathing for Life Trial;

Supplementary Table 2: Univariable analysis showing tidal breathing parameters in infants with and without bronchiolitis hospitalisation in the first year of life

	**Non-hospitalisation (n=1114)**	**Hospitalisation (n=89)**	**p-value†**
**Volumes and Respiratory Rate**			
TV, ml	7.38 (1.30)	7.22 (1.31)	0.257
Minute ventilation, mL/min	1.45 (0.27)	1.47 (0.28)	0.680
RR, min*	45.1 (10.4)	45.2 (10.5)	0.943
**Ratios**			
tPTEF/tE, % *	32.9 (13.6)	29.3 (16.3)	0.063
MTIF/MTEF	**1.30 (0.25)**	**1.38 (0.26)**	**0.006**
PTIF/PTEF	1.23 (0.25)	1.28 (0.25)	0.061
tE/tI	**0.80 (0.15)**	**0.76 (0.13)**	**0.020**

Data are presented as mean (SD)/* data are presented median (IQR). A t-test or Mann–Whitney test was used to compare groups. P-values <0.05 are shown in bold. TV, tidal volume; V'E, minute ventilation; RR, respiratory rate; tPTEF/tE, time to peak tidal expiratory flow divided by total expiratory time; MTIF/MTEF, ratio of medium-term inspiratory flow to medium-term expiration flow; PTIF/PTEF, ratio of time to peak tidal inspiratory flow to peak tidal expiratory flow, tI/tE the ratio inspiratory time to expiratory time. aOR, adjusted odds ratio; CI, confidence interval.

Supplementary Table 3. Logistic regression analysis bronchiolitis odds ratio per unit increase in lung function parameters.

	OR (95% CI)	p-value	aOR (95% CI)	p-value
**Volumes and Respiratory Rate**				
TV, mL	1.00 (0.98–1.05)	0.454	0.97 (0.80–1.17)	0.727
Minute ventilation, min/mL	1.00 (1.00-1.00)	0.417	1.00 (1.00–1.00)	0.647
RR, min	1.00 (0.97-1.02)	0.942	1.00 (0.98–1.02)	0.917
**Ratios**				
tPTEF/tE, %	1.00 (0.98-1.02)	0.058	0.99 (0.97–1.02)	0.586
MTIF/MTEF	**2.90 (1.12 – 7.51)**	**0.024**	**2.90 (1.20–7.02)**	**0.018**
PTIF/PTEF	**2.64 (1.01-6.94)**	**0.048**	**2.50 (1.02–6.12)**	**0.046**
tI/tE	**0.15 (0.03 – 0.95)**	**0.019**	**0.17 (0.03–0.87)**	**0.033**

Multivariable analysis adjusted for hospital admission due to bronchiolitis. P-values <0.05 are shown in bold.
TV, tidal volume; V'E, minute ventilation; RR, respiratory rate; tPTEF/tE, time to peak tidal expiratory flow divided by total expiratory time; MTIF/MTEF, ratio of medium-term inspiratory flow to medium-term expiration flow; PTIF/PTEF, ratio of time to peak tidal inspiratory flow to peak tidal expiratory flow, tI/tE the ratio inspiratory time to expiratory time. aOR, adjusted odds ratio; CI, confidence interval.

Supplementary Table 4: Univariable analysis showing tidal parameters of infants with and without asthma in childhood.

	**Non-asthma (n=594)**	**Asthma (n=100)**	**p-value**
**Volumes and respiratory rate**			
TV, ml/kg	7.42 (1.3)	7.21 (1.31)	0.132
Minute ventilation, L/min/kg	1.44 (0.3)	1.49 (0.3)	0.138
RR, min*	44.4 (13.7)	43.1 (15.0)	0.650
**Ratios**			
tPTEF/tE, %*	34.0 (14.3)	29.8 (12.2)	**<0.001**
MTIF/MTEF	1.30 (0.2)	1.41 (0.3)	**<0.001**
PTIF/PTEF	1.23 (0.2)	1.34 (0.3)	**<0.001**
tE/tI	0.80 (0.13)	0.75 (0.13)	**0.001**

Data presented as mean (SD)/* Values are presented as median (IQR). A t-test was used to compare groups. P-values <0.05 are shown in bold.
TV, tidal volume; V'E, minute ventilation; RR, respiratory rate; tPTEF/tE, time to peak tidal expiratory flow divided by total expiratory time; MTIF/MTEF, ratio of medium-term inspiratory flow to medium-term expiration flow; PTIF/PTEF, ratio of time to peak tidal inspiratory flow to peak tidal expiratory flow, tI/tE the ratio of inspiratory time to expiratory time.

Supplementary Table 5. Multivariable logistic regression analysis of the odds of having asthma per unit increase in infant lung function parameters.

	**Combined (n=694 total, n=100 asthma)**			
	**OR (95% CI)**	**p-value**	**aOR (95% CI)**	**p-value**
**Volumes and Respiratory Rate**				
TV, ml	**1.04 (1.01-1.07)**	**0.014**	1.03 (0.85–1.24)	0.785
Minute ventilation, L/min	1.00 (1.00 -1.00)	0.076	1.00 (0.99–1.00)	0.472
RR, min	1.00 (0.98 -1.02)	0.759	0.99 (0.97–1.02)	0.870
**Ratios**				
tPTEF/tE, %	**0.96 (0.94-0.98)**	**<0.0001**	**0.97 (0.94–0.99)**	**0.013**
MTIF/MTEF, %	**5.95 (2.64-13.4)**	**<0.0001**	**2.94 (1.07–8.02)**	**0.036**
PTIF/PTEF, %	**4.90 (2.19 – 10.8)**	**<0.0001**	**3.00 (1.11–8.15)**	**0.031**
tI/tE, %	**4.10 (1.21 – 13.9)**	**0.001**	0.18 (0.03–1.19)	0.072

Multivariable analysis adjusted for hospital admission or ED presentation due to bronchiolitis, maternal asthma during pregnancy, male sex, current breastfeeding at the time of lung function testing, and age at test. p values <0.05 are shown in bold.

ED, emergency department; TV, tidal volume; V'E, minute ventilation; RR, respiratory rate; tPTEF/tE, time to peak tidal expiratory flow divided by total expiratory time; MTIF/MTEF, ratio of medium-term inspiratory flow to medium-term expiration flow; PTIF/PTEF, ratio of time to peak tidal inspiratory flow to peak tidal expiratory flow, tI/tE the ratio inspiratory time to expiratory time. aOR, adjusted odds ratio; CI, confidence interval.

Supplementary Figures

Supplementary Figure 1: Graphical representation of the mediation models. Path (a) examines the relationship between asthma during pregnancy and infant lung function while controlling for covariates. Path (b) examines the relationship between infant lung function and bronchiolitis or asthma in childhood, while controlling for covariates. Path (c’, direct effect) examines the relationship between asthma during pregnancy and bronchiolitis or asthma in childhood, while controlling for covariates and for lung function prior to bronchiolitis episode. The indirect effect is calculated as a × b. The total effect is represented as c=c’ + a×b).

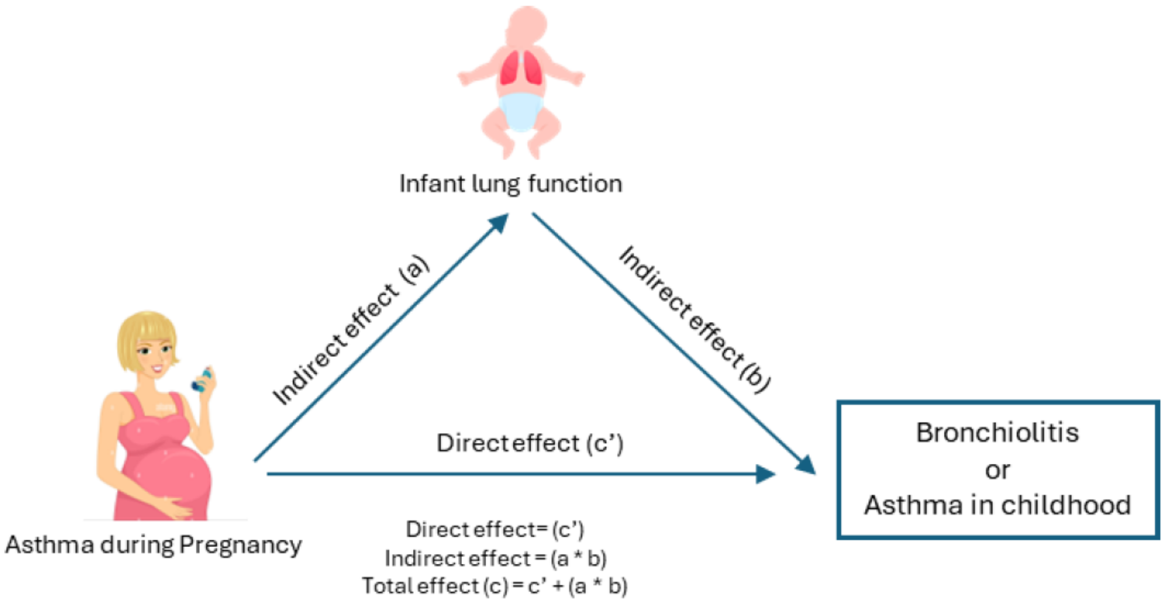

Supplementary Figure 2: Flow diagram illustrating the inclusion and exclusion criteria for the cohort studies:

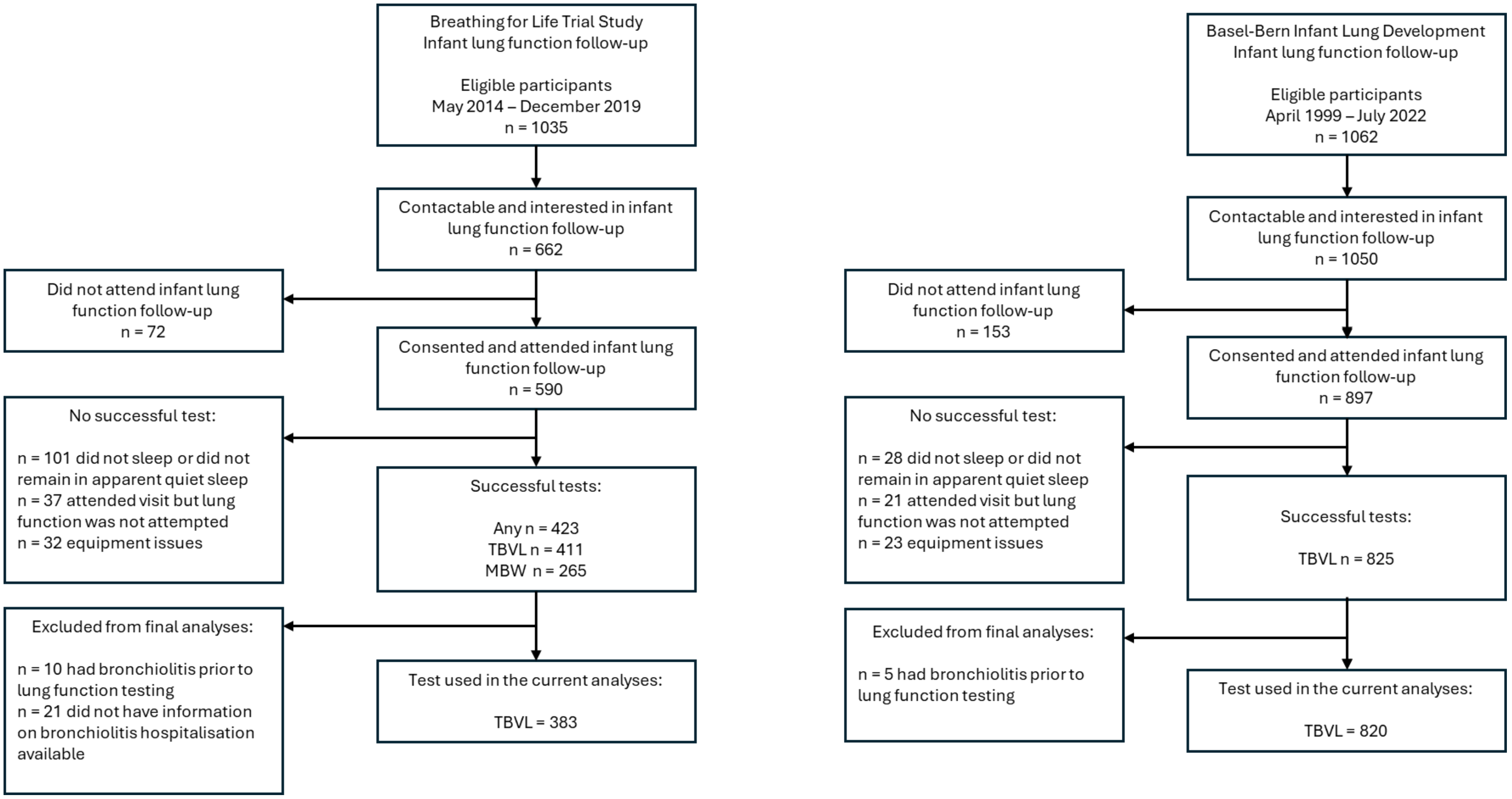

Supplementary Figure 3: Representative tidal breathing flow-volume curves from infants with and without bronchiolitis hospitalisation in the first year of life

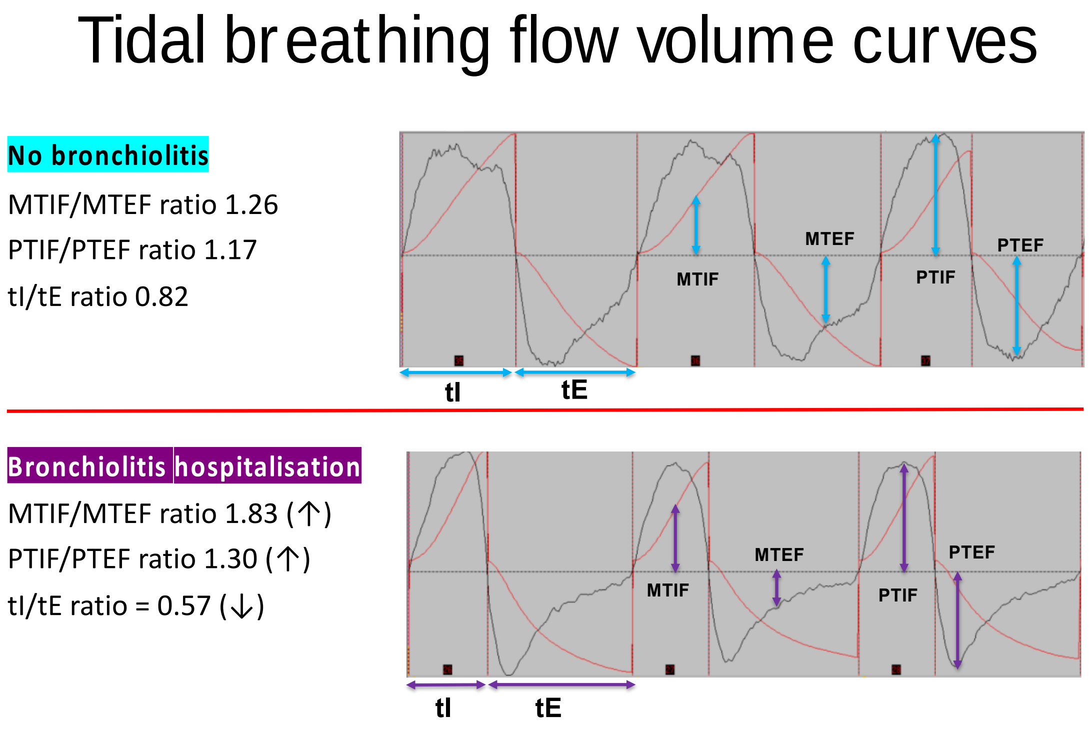

Footnote: Red line shows volume; black line shows flow

Supplementary Figure 4: Correlation between tidal breathing parameters

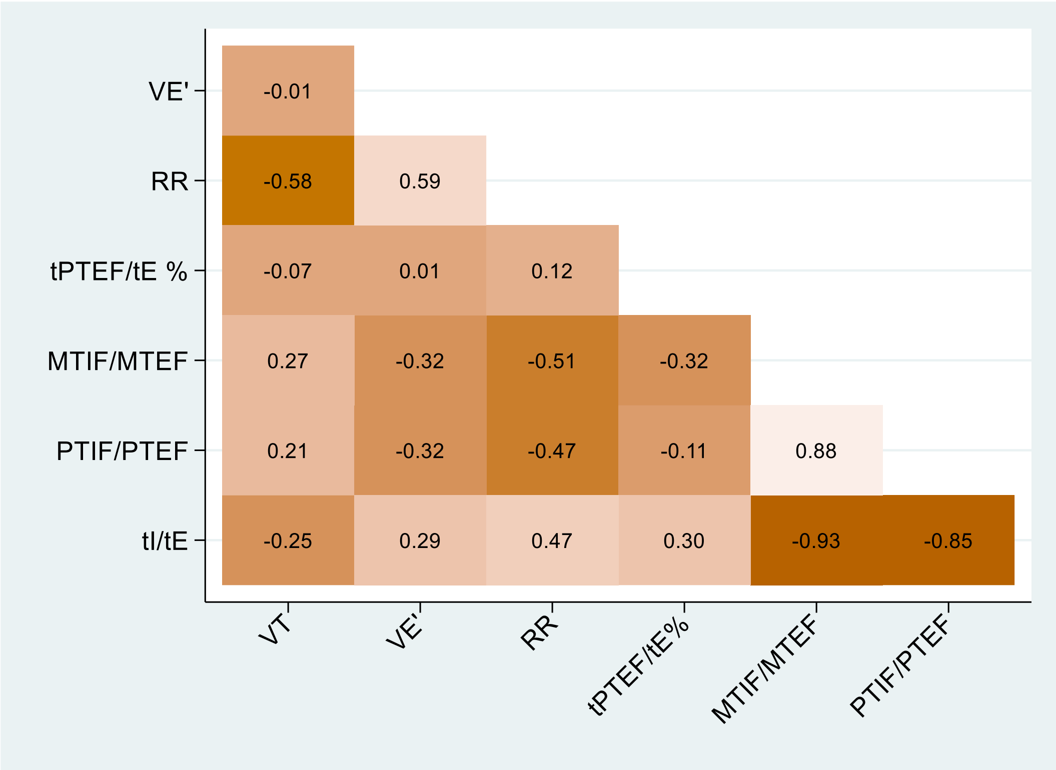

Correlation coefficients (r) were calculated using Pearson’s correlation.

References

1. Murphy, V.E., et al., *The Breathing for Life Trial: a randomised controlled trial of fractional exhaled nitric oxide (FENO)-based management of asthma during pregnancy and its impact on perinatal outcomes and infant and childhood respiratory health.* BMC Pregnancy and Childbirth, 2016. **16**(1): p. 111.
2. Murphy, V.E., et al., *Effect of asthma management with exhaled nitric oxide versus usual care on perinatal outcomes.* Eur Respir J, 2022. **60**(5).
3. de Gouveia Belinelo, P., et al., *Maternal asthma is associated with reduced lung function in male infants in a combined analysis of the BLT and BILD cohorts.* Thorax, 2021. **76**(10): p. 996-1001.
4. Fuchs, O., et al., *Cohort profile: the Bern infant lung development cohort.* Int J Epidemiol, 2012. **41**(2): p. 366-76.
5. Salem, Y., et al., *Cohort Profile Update: The Bern Basel Infant Lung Development Cohort.* Int J Epidemiol, 2024. **53**(1).
6. Bates, J.H., et al., *Tidal breath analysis for infant pulmonary function testing. ERS/ATS Task Force on standards for infant respiratory function testing. European Respiratory Society/American Thoracic Society.* Eur Respir J, 2000. **16**(6): p. 1180-92.
7. Frey, U., et al., *Specifications for equipment used for infant pulmonary function testing. ERS/ATS Task Force on Standards for Infant Respiratory Function Testing. European Respiratory Society/ American Thoracic Society.* Eur Respir J, 2000. **16**(4): p. 731-40.
8. Latzin, P., et al., *Elevated exhaled nitric oxide in newborns of atopic mothers precedes respiratory symptoms.* Am J Respir Crit Care Med, 2006. **174**(12): p. 1292-8.
9. Miller, M.R., et al., *Standardisation of spirometry.* Eur Respir J, 2005. **26**(2): p. 319-38.
10. Asher, M.I., et al., *International Study of Asthma and Allergies in Childhood (ISAAC): rationale and methods.* Eur Respir J, 1995. **8**(3): p. 483-91.
11. Graham, B.L., et al., *Standardization of Spirometry 2019 Update. An Official American Thoracic Society and European Respiratory Society Technical Statement.* Am J Respir Crit Care Med, 2019. **200**(8): p. e70-e88.
12. Decrue, F., et al., *Increased Impact of Air Pollution on Lung Function in Preterm versus Term Infants: The BILD Study.* Am J Respir Crit Care Med, 2022. **205**(1): p. 99-107.
13. Da Silva Sena, C.R., et al., *Higher exhaled nitric oxide at 6 weeks of age is associated with less bronchiolitis and wheeze in the first 12 months of age.* Thorax, 2022. **77**(11): p. 1106-1112.
14. Gunzler, D., et al., *Introduction to mediation analysis with structural equation modeling.* Shanghai Arch Psychiatry, 2013. **25**(6): p. 390-4.
15. Mehmetoglu, M., *Medsem: a Stata package for statistical mediation analysis.* Int. J. Computational Economics and Econometrics, 2018. **8**(1): p. 63–78.
